# Acute Perioperative Comparison of Patient-Specific Instrumentation versus Conventional Instrumentation Utilization during Bilateral Total Knee Arthroplasty

**DOI:** 10.1155/2018/9326459

**Published:** 2018-02-21

**Authors:** Jerrod A. Steimle, Michael T. Groover, Brad A. Webb, Brian J. Ceccarelli

**Affiliations:** Department of Orthopedics, Grandview Medical Center, Affiliate of Kettering Medical Center and Ohio University Heritage College of Osteopathic Medicine, 405 W. Grand Ave., Dayton, OH 45405, USA

## Abstract

Utilizing patient-specific instrumentation during total knee arthroplasty has gained popularity in recent years with theoretical advantages in blood loss, intraoperative time, length of stay, postoperative alignment, and functional outcome, amongst others. No study has compared acute perioperative measures between patient-specific instrumentation and conventional instrumentation in the bilateral total knee arthroplasty setting. We compared patient-specific instrumentation versus conventional instrumentation in the setting of bilateral total knee arthroplasty to determine any benefits in the immediate perioperative period including surgical time, blood loss, pain medication use, length of stay, and discharge disposition. A total of 49 patients with standard instrumentation and 31 patients with patient-specific instrumentation were retrospectively reviewed in a two-year period at one facility. At baseline, the groups were comparable with respect to age, ASA, BMI, and comorbid conditions. We analyzed data on operative time, blood loss, hemoglobin change, need for transfusion, pain medication use, length of stay, and discharge disposition. There was no statistically significant difference between groups in regards to these parameters. Patient-specific instrumentation in the setting of bilateral total knee arthroplasty did not provide any immediate perioperative benefit compared to conventional instrumentation.

## 1. Introduction

Total knee arthroplasty (TKA) is a common orthopedic procedure used to treat osteoarthritis of the knee. Of patients undergoing TKA, approximately one-third will have bilateral joint disease [1]. Because of this, patients and surgeons are faced with the difficult decision of determining whether it is most beneficial for the patient to perform simultaneous bilateral TKA versus staged bilateral TKA. Proposed advantages of simultaneous bilateral TKA include limiting surgery, anesthesia, rehabilitation, and hospital stay to a single event, translating to reduced cost to the patient and to the hospital, and a quicker overall patient recovery [2]. However, there are well-defined risks associated with simultaneous bilateral TKA, and the decision to pursue this should be made with the patient's best interests in mind [3–5]. One proposed method of reducing perioperative morbidity is the utilization of patient-specific instrumentation (PSI) rather than standard instrumentation during the surgical procedure. This has the theoretical advantages of reduced blood loss, reduced operative time, avoidance of systemic emboli (due to the avoidance of intramedullary guide-rod placement), improved preoperative planning with additional information gleaned from advanced imaging, and improvement in mechanical alignment.

PSI first became popularized as an alternative to computer-assisted TKA to allow for more predictable alignment and theoretical improvement in long-term implant survival. Computer-assisted TKA requires increased operating costs and increased surgical time due to system setup, results in difficulty registering bony landmarks and fractures from pin insertion, and requires a learning curve to appropriately understand the protocol [6]. PSI aimed to achieve the same goals laid out by computer-assisted TKA, while shifting the work of computer navigation from the intraoperative setting to the preoperative period [7]. Currently, the process of providing data to the manufacturers of the PSI includes obtaining anatomical parameters utilizing magnetic resonance imaging (MRI) or computer tomography (CT) scans [8].

The results of unilateral TKA utilizing PSI have been mixed. While some studies show improved alignment with PSI postoperatively, the consensus appears to be that PSI does not improve the accuracy of alignment of the components in TKA compared with conventional instrumentation [9, 10]. In terms of operative time, the results are inconclusive. Some studies cite quicker surgical times, while others cite no difference or even longer surgical times [11–15]. The results are also inconsistent in terms of intraoperative blood loss and length of stay [7, 16–21]. The patient outcome data are limited. Implant survival or patient satisfaction has not yet been assessed.

There are limited data available regarding PSI in the bilateral total knee arthroplasty setting. This retrospective review aims to determine the advantages associated with PSI in the bilateral TKA setting.

## 2. Materials and Methods

The medical records of patients who underwent simultaneous bilateral total knee arthroplasty at an urban teaching hospital in the Midwest between July 8, 2013, and July 8, 2015, were reviewed. Using reporting functions available in electronic medical records, charts were identified using ICD9 715.9x (osteoarthritis of the bilateral knees) and CPT 27447 (bilateral total knee arthroplasty). Degree of deformity was unable to be discerned due to the retrospective nature of the review.

Inclusion criteria included patients with the above diagnosis and procedural codes, aged ≥ 18 or ≤89, without any previous knee arthroplasty procedures. Exclusion criteria included patients aged < 18 or >89, a diagnosis of rheumatoid arthritis, posttraumatic arthritis or inflammatory arthritis of the knee (ICD9 714.x), previous total knee arthroplasty procedure, and patients undergoing revision arthroplasty or patients undergoing unicompartmental or patellofemoral joint arthroplasty. A total of 91 bilateral knee arthroplasty procedures were performed between the studied time period. Of those, a total of 80 patients were included in the study who met the inclusion criteria and did not exhibit any exclusion criteria. The remaining 11 patients were excluded secondary to either undergoing bilateral patellofemoral arthroplasty and bilateral unicompartmental arthroplasty or had a diagnosis of rheumatoid, posttraumatic or inflammatory arthritis.

Two groups were created using the above parameters. Group 1 included all patients who met the inclusion criteria while not exhibiting any exclusion criteria between the studied time period and had the procedure performed using standard instrumentation. Group 1 included a total of 49 patients. Group 2 included all patients who met the inclusion criteria while not exhibiting any exclusion criteria between the studied time period and had the procedure performed using PSI. Group 2 included a total of 31 patients.

The charts were reviewed according to the following outcomes: surgical time (defined by time of incision to skin closure), tourniquet time (defined by time just prior to incision to implant placement and cement bonding), blood loss (assessed via anesthesia department by suction fluid volume during the case), change in hemoglobin up to 48 hours postoperatively, number of units of packed red blood cells transfused, morphine milligram equivalent used, length of stay, and discharge disposition. The calculation for morphine milligram equivalent used up to 24 hours post-op and from 24 to 48 hours post-op including morphine, dilaudid, demerol, oxycodone, hydrocodone, oxycontin, and tramadol is outlined by Von Korff et al. [22]. Demographic or personal characteristics analyzed included age at the time of surgery, comorbidities, ASA category, and BMI.

These surgeries were performed by a cohort of five practicing attending orthopedic surgeons with high volume in total knee arthroplasty (defined by >70 procedures per year), utilizing a medial parapatellar approach with identical multimodal pain management protocols with the exception of the utilization of a periarticular injection of ropivacaine with epinephrine or liposomal bupivacaine in only 48/80 patients (31 standard and 17 PSI), solely based on surgeon preference [23]. Surgeon 1 was involved with thirty-two simultaneous bilateral TKAs (18 standard and 14 PSI). All thirty-two of this surgeon's cases were performed with surgeon 2 operating simultaneously on their respective sides. Surgeon 2 was involved with fifty-seven simultaneous bilateral TKAs (28 standard and 29 PSI). Twenty-five of this surgeon's cases were performed alone, one side at a time (10 standard and 15 PSI), and thirty-two were performed with surgeon 1 (18 standard and 14 PSI) operating simultaneously on their respective sides. Surgeon 3 and surgeon 4 were involved with seventeen simultaneous bilateral TKAs performed together simultaneously on their respective sides (15 standard and 2 PSI). Surgeon 5 was involved with six simultaneous bilateral TKAs performing one side at a time, all of those being standard instrumentation.

PSI utilized was MRI-based cutting jigs defined from the patient's anatomy based on preoperative MRI. Implants used in these cases were from the Zimmer Persona or Natural system (Warsaw, IN). Implants used in all other cases were off-the-shelf Stryker Triathlon (Kalamazoo, MI), Zimmer Persona, or Zimmer Natural systems. Each patient received identical posterior stabilized cemented implants between the right and left sides, without the use of stems or augments. The decision to utilize Stryker or Zimmer implants in the standard instrumentation group was made by surgeon preference.

The study was designed to have 80% power to detect a 0.6 standard deviation difference between the groups, resulting in 30 patients per group to achieve statistical significance at alpha 0.05, two-tailed, using an independent *t*-test.

## 3. Results

The comparison of demographic information and personal characteristics of the two groups was similar (Table 1), including age, comorbidities, ASA category, and BMI. None of these parameters were statistically significant.

Table 2 details the immediate perioperative measures including surgical time, tourniquet time, blood loss, change in hemoglobin up to 48 hours postoperatively, number of units of packed red blood cells transfused, morphine milligram equivalent used, length of stay, and discharge disposition. Virtually, all of these parameters were similar across both groups without any statistical significance.

Length of surgery from incision to closure of both incisions was similar between the two groups (99 minutes for standard and 102.2 minutes for PSI). Average tourniquet time was also similar (60.3 for standard and 62.7 for PSI).

Intraoperative blood loss was slightly less for standard instrumentation, 253.8 mL, versus PSI, 275 mL. The change in hemoglobin from preoperative measurements to postoperative day 1 and then to postoperative day 2 was similar as well. These values were 3.14 and 1.31 for standard instrumentation and 3.07 and 1.14 for PSI, respectively. The need for blood transfusion was also comparable. 46.94% of the standard instrumentation group and 48.39% of the PSI group did not require a transfusion. Of those requiring a transfusion, 4.08% of the standard group and 3.23% of the PSI group required only 1 unit of PRBCs. 38.78% of the standard group and 45.16% of the PSI group required 2 units of PRBCs. Interestingly, 10.20% of the standard group required >2 units of PRBCs, while only 3.23% of the PSI group required >2 units of PRBCs. The decision to transfuse was based on a combination of clinical findings (shortness of breath, light headedness, dizziness, etc.) with a hemoglobin concentration of <8.0 g/dL. No specific cutoff for hemoglobin or hematocrit was used as sole determinants to transfuse.

Mean morphine milligram equivalent was nearly identical between the two groups for the periods from surgery to 24 hours after surgery, from 24 to 48 hours from surgery, and for the entire 48 hour period. The MME for the entire 48 hours postoperatively for the standard group was 68.7 mg and was 66.1 for the PSI group.

The average length of stay between the two groups was 4.5 days for the standard group and 4.4 days for the PSI group. The proportion of patients discharging in less than 3 days, between 3 and 5 days, and greater than 5 days was also comparable across the board.

In regards to discharge disposition, 55.1% of patients in the standard group were discharged home, while only 32.26% of the patients in the PSI group were discharged home. 28.57% of standard and 25.81% PSI were discharged to inpatient rehab. Only 16.3% of patients in the standard group required a skilled nursing facility, while 41.94% of the patients in the PSI group required a skilled nursing facility.

Of note, there were no major acute perioperative complications including heart attack, stroke, periprosthetic fracture, acute wound complications, or infection, amongst others.

## 4. Discussion

Given the increasing number of joint replacement procedures performed, in particular, total knee arthroplasty, newer innovations have been developed with the aspiration to increase efficiency and improve outcomes. These innovations include, but are not limited to, computer-assisted navigation, patient-specific instrumentation, and patient-specific custom implants. Studies have been performed comparing these newer modalities with standard procedures, but none have been performed in the setting of bilateral total knee arthroplasty. Given the inconsistent results regarding these newer advances in technology compared to standard procedures in the unilateral arthroplasty setting, our aim was to determine the immediate perioperative benefit of patient-specific instrumentation compared to standard instrumentation.

In terms of operative time, one study cited an overall surgical time average of 6.7 minutes shorter with PSI, while another cited 13 minutes shorter with PSI [11, 12]. On the contrary, Hamilton et al. demonstrated that PSI was on average 4 minutes longer than standard instrumentation in terms of surgical time [14]. There was no statistically significant difference between surgical time in our study between the two groups. While patient-specific instrumentation in theory would provide a quicker surgical time due to custom cutting blocks and the lack of need for adjustment, this has not been demonstrated throughout the literature. Additionally, preoperative prep work may be increased with PSI due to learning curve, understanding of PSI instrumentation, and reviewing preoperative templates to ensure appropriate bony resection, implant alignment, and sizing.

Noble et al. also reported a reduction in inpatient length of stay as well as intraoperative blood loss using PSI for unilateral TKA. His findings suggest reduced blood loss and shorter length of stays for PSI [11]. However, other studies contradict these findings. The Abane et al. study failed to demonstrate any statistically significant difference amongst intraoperative blood loss and length of stay between conventional instrumentation and PSI [19]. Our results agree with the Abane et al. study, with no statistically significant difference amongst intraoperative blood loss or length of stay.

Limited data exist in regards to pain control utilizing PSI. In this study, we found no statistically significant difference amongst PSI and conventional instrumentation. Mean morphine equivalent use was essentially equivalent for both groups.

While the immediate perioperative measures in this study fail to identify a definitive advantage of PSI over conventional instrumentation, there is a theoretical advantage in terms of cost analysis of utilizing PSI. However, a recent study by Barrack et al. failed to identify a cost benefit in regards to utilizing PSI over conventional instrumentation. His study identified that while the cutting guides had significantly lower total operative time and instrument processing time, the estimated $322 savings was overwhelmed by a $1,500 additional cost of the MRI and the PSI cutting guide [21, 24]. Nunley et al. also failed to identify a cost-effective reason to utilize PSI over conventional instrumentation [25].

One limitation to this study is the lack of outcomes involving functionality, alignment, or patient satisfaction. While some studies show improved alignment in the unilateral setting with PSI postoperatively, the general consensus appears to be that PSI does not improve the accuracy of alignment of the components in TKA compared with conventional instrumentation [9, 10]. In addition, one study looked at the range of motion at one year postoperatively in regards to PSI versus standard instrumentation and found that the PSI group had on average 3.9 degrees of less range of motion [23]. Another limitation was the fact that five different surgeons were used in this retrospective review. While all five are high-volume surgeons in regards to total knee arthroplasty, they certainly provide variation in technique. Moreover, in 49/80 patients, each side was performed simultaneously with two surgeons at the same time, while 31/80 patients had a single surgeon perform one side at a time. Because of this, thirty-one of the patients had longer times under anesthesia, which can theoretically contribute to variations in the results. Additionally, only 48/80 patients received a periarticular injection of either ropivacaine with epinephrine or liposomal bupivacaine, based on surgeon preference (31 standard and 17 PSI). Again, this can certainly impact postoperative pain medication usage.

## 5. Conclusion

While new innovations are being developed to provide a more efficient, cost effective, and outcome driven improvement in total knee arthroplasty, this study failed to identify any advantages to PSI in the bilateral total knee setting in regards to immediate perioperative measures. Certainly, there exist theoretical advantages that are difficult to quantify, and additional studies may provide a better understanding of these.

## Figures and Tables

**Table 1:** Demographic characteristics between groups.

Category	Standard (*N* = 49)	PSI (*N* = 31)	*p* value
*Age (years), mean (SD)*	61.0 (9.9)	60.8 (8.5)	0.898
<50	8 (16.33%)	3 (9.68%)	—
50–65	25 (51.02%)	20 (64.52%)	—
>65	16 (32.65%)	8 (25.81%)	—
*BMI (kg/m* ^*2*^ *), mean*	33.6	34.9	0.423
<30	20 (40.82%)	6 (19.35%)	—
30–35	9 (18.38%)	13 (41.94%)	—
>35	20 (40.82%)	12 (38.71%)	—
*ASA*, *mean*	2.5	2.3	0.215
1	1 (2.04%)	0 (0%)	—
2	24 (48.98%)	21 (67.74%)	—
3	24 (48.98%)	10 (32.36%)	—
*Number of comorbidities* ^∗^			0.229
0-1	18 (36.73%)	15 (48.39%)	—
2-3	24 (48.98%)	15 (48.39%)	—
>3	7 (14.29%)	1 (3.23%)	—

^∗^Comorbidities included in this study: Hyperlipidemia, hypertension, heart disease, cancer history, chronic kidney disease, diabetes mellitus, thyroid disease, chronic obstructive pulmonary disease, hepatitis, and pancreatitis; *N* = sample size; SD = standard deviation.

**Table 2:** Perioperative outcomes between groups.

Category	Standard (*N* = 49)	PSI (*N* = 31)	*p* value
Total length of surgery from incision to closure (min)	99 (21.3)	102.2 (13.4)	0.721
*Tourniquet time (min), mean (SD)*			
Total	60.3 (16.1)	62.7 (10.5)	0.486
Left	57.8 (15.4)	57.4 (12.2)	0.906
Right	62.8 (19.5)	67.9 (13.6)	0.020^∗^
Intraoperative blood loss (mL), mean (SD)	253.8	275	0.530
*Change in Hg (g/dL)* ^*1*^ *, mean (SD)*			
Pre-op to POD1	3.14 (1.31)	3.07 (0.99)	0.788
POD1 to POD2	0.97 (1.18)	0.62 (1.28)	0.221
*Units of blood transfused (units of PRBCs)*	1.2	1.03	0.577
0 units	23 (46.94%)	15 (48.39%)	—
1 unit	2 (4.08%)	1 (3.23%)	—
2 units	19 (38.78%)	14 (45.16%)	—
>2 units	5 (10.20%)	1 (3.23%)	—
*Morphine milligram equivalent (mg), mean (SD)*			
Up to 24 hours post-op	35.8 (14.8)	37.8 (12.5)	0.543
24–48 hours post-op	32.7 (15.1)	28.3 (15.3)	0.208
Entire 48-hour period	68.7 (27.2)	66.1 (25.5)	0.667
*Length of stay including surgery day (days), mean (SD)*	4.5 (1.4)	4.4 (1.3)	0.611
<3	7 (14.29%)	6 (19.35%)	—
3–5	35 (71.43%)	20 (64.52%)	—
>5	7 (14.29%)	5 (16.13%)	—
*Discharge disposition*			0.450
Home	14 (28.60%)	10 (32.26%)	—
Inpatient rehab facility	8 (16.30%)	8 (25.81%)	—
Skilled nursing facility	27 (55.10%)	13 (41.94%)	—

^1^Excluding values that increased after a transfusion. ^∗^Nonparametric statistics, specifically Mann–Whitney *U* test was used, for statistical significance.
